# Contrasting effects of plant inter‐ and intraspecific variation on community trait responses to restoration of a sandy grassland ecosystem

**DOI:** 10.1002/ece3.2711

**Published:** 2017-01-24

**Authors:** Xiaoan Zuo, Xiyuan Yue, Peng Lv, Qiang Yu, Min Chen, Jing Zhang, Yongqing Luo, Shaokun Wang, Jing Zhang

**Affiliations:** ^1^ Northwest Institute of Eco‐Environment and ResourcesChinese Academy of SciencesLanzhouChina; ^2^ Laboratory of Stress Ecophysiology and Biotechnology (LSEB)NIEER, CASLanzhouChina; ^3^ National Hulunber Grassland Ecosystem Observation and Research StationInstitute of Agricultural Resources and Regional PlanningChinese Academy of Agricultural SciencesBeijingChina

**Keywords:** Community weighted traits, functional traits, interspecific and intraspecific variation, sandy grassland ecosystem, soil gradient, vegetation restoration

## Abstract

Changes in plant community traits along an environmental gradient are caused by interspecific and intraspecific trait variation. However, little is known about the role of interspecific and intraspecific trait variation in plant community responses to the restoration of a sandy grassland ecosystem. We measured five functional traits of 34 species along a restoration gradient of sandy grassland (mobile dune, semi‐fixed dune, fixed dune, and grassland) in Horqin Sand Land, northern China. We examined how community‐level traits varied with habitat changes and soil gradients using both abundance‐weighted and non‐weighted averages of trait values. We quantified the relative contribution of inter‐ and intraspecific trait variation in specific leaf area (SLA), leaf dry matter content (LDMC), leaf carbon content (LCC), leaf nitrogen content (LNC), and plant height to the community response to habitat changes in the restoration of sandy grassland. We found that five weighted community‐average traits varied significantly with habitat changes. Along the soil gradient in the restoration of sandy grassland, plant height, SLA, LDMC, and LCC increased, while LNC decreased. For all traits, there was a greater contribution of interspecific variation to community response in regard to habitat changes relative to that of intraspecific variation. The relative contribution of the interspecific variation effect of an abundance‐weighted trait was greater than that of a non‐weighted trait with regard to all traits except LDMC. A community‐level trait response to habitat changes was due largely to species turnover. Though the intraspecific shift plays a small role in community trait response to habitat changes, it has an effect on plant coexistence and the maintenance of herbaceous plants in sandy grassland habitats. The context dependency of positive and negative covariation between inter‐ and intraspecific variation further suggests that both effects of inter‐ and intraspecific variation on a community trait should be considered when understanding a plant community response to environmental changes in sandy grassland ecosystems.

## 1 Introduction

The study of how plant communities are assembled in the context of environmental changes is crucial for explaining species coexistence and biodiversity maintenance (Bhaskar, Dawson, & Balvanera, 2014; Mason et al., 2012; Sutherland et al., 2013). Numerous studies have suggested that plant community assembly is driven by environmental filtering, which can be explained by the associations of plant functional traits with environment changes (Bernard‐Verdier et al., 2012; Diaz, Cabido, & Casanoves, 1998; Marteinsdottir & Eriksson, 2014; Pillar, Duarte, Sosinski, & Joner, 2009; Vilà‐Cabrera, Martínez‐Vilalta, & Retana, 2015). The trait‐based approach shows that plant functional traits are some of the most reliable predictors of ecological properties of plant communities and their responses to environmental changes (Huang et al., 2016; Lavorel, 2013; Lienin & Kleyer, 2012). Changes of plant community‐level traits along the environment gradient are mainly caused by interspecific trait variation, intraspecific trait variation, or a combination of these two (Kichenin, Wardle, Peltzer, Morse, & Freschet, 2013; Lepš, de Bello, Šmilauer, & Doležal, 2011). To examine the role of interspecific and intraspecific trait variation in plant community responses to environmental change is very important for understanding the underlying mechanisms of plant community assembly (Bhaskar et al., 2014; Vilà‐Cabrera et al., 2015).

Vegetation succession can be regarded as plant community assembly in an ecological process and therefore provide an ideal setting to test whether functional traits are important for plant community assembly (Schleicher, Peppler‐Lisbach, & Kleyer, 2011; Zhang et al., 2015). The trait‐based approach has often been applied in vegetation succession of forestry ecosystems (Bhaskar et al., 2014; Lohbeck et al., 2013; Mason et al., 2012; Zhang et al., 2015), providing valuable insight into plant community assembly. However, very few studies have used the functional traits in vegetation succession of natural grassland ecosystems (Marteinsdottir & Eriksson, 2014).

The environmental filtering effect suggests that species having functional traits within a certain range can succeed in establishing a community in the given environmental conditions (Diaz et al., 1998; Jung, Violle, Mondy, Hoffmann, & Muller, 2010). Numerous studies focus on the effect of interspecific trait variation on community assembly (Garnier et al., 2001; Lohbeck et al., 2014; McGill, Enquist, Weiher, & Westoby, 2006) because differences in trait values are much larger among species (Kichenin et al., 2013; Le Bagousse‐Pinguet, de Bello, Vandewalle, Leps, & Sykes, 2014). Recently, a growing number of studies have demonstrated that the intraspecific trait variation also plays an important role in structuring a plant community (Carlucci, Debastiani, Pillar, & Duarte, 2015; Kichenin et al., 2013; Laughlin, Joshi, van Bodegom, Bastow, & Fule, 2012; Siefert, 2012; Vilà‐Cabrera et al., 2015). The intraspecific trait variation may improve species selection in environmental filtering by adjusting species trait values to environment requirements, thus favoring the inclusion of species in a community due to the niche overlap among co‐occurring species (de Bello, Carmona, Mason, Sebastia, & Leps, 2013; Jung et al., 2010). So, a higher relative importance of intraspecific trait variation linked to environmental changes reflects the higher resistance of plant community structure to environmental filtering (Mason et al., 2012). However, studies examining the relative contribution of interspecific and intraspecific trait variation to the response of plant community traits in an environmental gradient in grassland ecosystems are still lacking.

Empirical studies have shown that the relative importance of interspecific trait variation can increase along a wide environmental gradient (Albert, Grassein, Schurr, Vieilledent, & Violle, 2011; Kichenin et al., 2013; Vilà‐Cabrera et al., 2015). In particular, the weighted community‐average values are determined by the functional traits of dominant species, while the non‐weighted community‐average values are determined by the presence–absence of a species in a community (Janeček et al., 2013; Violle et al., 2012). Increasing studies have shown that ecosystem function and properties are driven by traits of dominant species in a plant community (Butterfield & Suding, 2013; Finegan et al., 2015). The rapid shift of dominant species across habitat changes reveals that the interspecific variation is the important driver of changes in ecosystem processes (Vilà‐Cabrera et al., 2015; Zuo, Zhang, et al., 2016). Meanwhile, the competition for limited resources along a short environmental gradient tends to form the intraspecific trait variation of co‐occurring species, thereby promoting species coexistence and thus affecting community function (Auger & Shipley, 2013; Lepš et al., 2011; Mason et al., 2012). So, based on the weighted and non‐weighted community‐average trait values, understanding how a plant community responds to environmental changes through the interspecific or intraspecific trait variation can be seen as a first step toward linking environment changes to ecosystem function.

The Horqin sandy grassland is located in the semi‐arid area of southeastern Inner Mongolia, northern China. The original natural vegetation was a grass‐dominated steppe with scattered trees (mainly elms, *Ulmus* spp.), which has been replaced by sandy dunes and grasslands, due to long‐term overgrazing and fuel gathering (Zhao, Zhao, Zhou, Zhang, & Drake, 2005; Zhao, Zhou, Zhang, & Zhao, 2006). However, due to annual precipitation between 350 and 500 mm, mobile dunes can be stabilized and transformed to semi‐fixed and fixed dunes after excluding grazing for approximately 15 and 30 years, respectively (Li, Zhao, Chen, Luo, & Wang, 2012; Liu, Zhao, Zhao, Zuo, & Drake, 2009; Zhang, Zhao, Zhang, Zhao, & Drake, 2005). A classical vegetation succession occurs from the sand pioneer plant in the mobile dune to the low shrub communities in the semi‐fixed dune then toward the annual herb dominated communities in the fixed dune or grassland (Zhang et al., 2005; Zuo et al., 2009). Soil gradients related to organic carbon, total nitrogen, pH, electrical conductivity, very fine sand, silt, and clay strongly affect the distribution and composition of plant communities in the restoration of sandy grassland (Zuo et al., 2009). However, little is known about how plant community‐level traits respond to habitat changes in the restoration of sandy grasslands. Understanding the role of interspecific and intraspecific trait variation on plant community trait responses to habitat changes is helpful for vegetation restoration and land management in a sandy grassland ecosystem.

Comparative efforts focus on analyzing the interspecific and intraspecific variation due to habitat differentiation (Auger & Shipley, 2013; Hulshof et al., 2013; Kichenin et al., 2013); therefore, a key goal in our study is to examine the effects of interspecific and intraspecific variation on plant community‐average traits within the same framework. We ask two questions: how the community‐average trait responds to habitat changes or soil gradients in the restoration of sandy grassland; and to what extent does the interspecific or intraspecific trait variation contribute to community‐average trait responses to habitat changes? Specifically, we hypothesized that (1) interspecific variation will have a higher contribution than intraspecific variation to the effects of community‐average traits because of obvious vegetation succession across habitat changes; and (2) the relative contribution of interspecific trait variation is higher for abundance‐weighted than for non‐weighted measures of community‐level trait variation.

## 2 Materials and Methods

### 2.1 Site area

This study was conducted in a sandy grassland ecosystem of Horqin Sandy Land (42°55′ N, 120°42′ E; 360 m elevation) in the northeast part of Inner Mongolia, Northern China. The area has a strong temperate, semi‐arid continental monsoonal climate with a warm summer and a very cold winter. The mean annual precipitation is 360 mm, with more than 75% falling within a growing season from June to September. The mean annual temperature is approximately 6.4°C, with monthly mean temperatures ranging from a minimum of −16.8°C in January to a maximum of 23.5°C in July.

The landscape is composed of different sandy grasslands: mobile dunes, semi‐fixed dunes, fixed dunes, and grasslands (Liu, Zhao, & Zhao, 2009). Soils are primarily sandy chestnut soils and are vulnerable to wind erosion. Sandy grasslands are covered with the native forbs, grasses, and shrubs. Mobile dunes have vegetation cover of less than 10% and are dominated by the sand pioneer plant, annual forb of *Agriophyllum squarrosum*. Semi‐fixed dunes (10–60% vegetation cover) and fixed dunes (more than 60% vegetation cover) are dominated by the asexual reproductive shrub *Artemisia halodendron* and the annual forb of *Artemisia scoparia*, respectively. Grasslands have more than 60% vegetation cover and are dominated by the annual forb *A. scoparia* and perennial grasses *Phragmites communis and Pennisetum centrasiaticum*.

In early August 2013, we established 24 plots (20 × 20 m), 0.5–8 km apart, across a typical restoration gradient of sandy grassland, which included mobile dune (MD), semi‐fixed dune (SFD), fixed dune (FD), and grassland type (G) (Zuo, Zhang, et al., 2016; Zuo, Wang, et al., 2016). There are six replicate plots in each habitat type. Semi‐fixed dunes and fixed dunes were naturally restored from mobile dunes by fencing out livestock grazing from approximately 1995 to 1980. Before grazing exclusion, the landscape of these dune sites was characterized by areas with mobile dunes. Grassland sites were also protected by a fence to prevent grazing from 1996 to the present, representing the benchmark sites of sandy grassland conservation and restoration.

### 2.2 Vegetation composition and measurement

We set up five 1 × 1 m quadrats at the four corners and the center of each plot. Within each quadrat, we estimated vegetation cover, recorded the number of plant species, and then harvested aboveground biomass of each species during the peak of the growing season. Aboveground plant biomass was dried at 60°C for 48 hr and weighed in the lab. Thus, species abundance was calculated as the relative biomass of each species to the total biomass in the plot.

### 2.3 Soil measurement

Within the quadrat, three random soil samples were collected at a depth of 0–10 cm using a 3‐cm diameter soil auger. Soil samples were pooled and sieved (2‐mm mesh) for laboratory analysis. Soil water content in each quadrat was measured on an additional sample, which was dried to a constant weight at 60°C in the lab. Soil samples in each quadrat were also collected for soil bulk density using a soil auger equipped with a stainless‐steel cylinder (5 cm in both diameter and height).

Soil C and N contents were determined by an elemental analyzer (vario Macro cube, Elementar, Hanau, Germany). Soil pH and electrical conductivity (EC) were measured in a 1:1 soil water slurry and in a 1:5 soil water aqueous extract (Multiline F/SET‐3, WTW, Weilheim, Germany), respectively. Soil texture from international and USDA classification systems was determined by the wet sieving method (Li, Awada, et al., 2012). Data from five quadrats were averaged to allow us to estimate vegetation characteristics and soil properties in each plot. Vegetation characteristics and soil properties at four different habitats are summarized in Table S1.

Soil properties from 24 plots were converted into the aggregate variables via the principal component analyses (PCA) along the restoration gradient of sandy grassland (Zuo, Wang, et al., 2016). The first component explained over 86% of the total variability and was regarded as the main soil gradient (Table S2). Hereafter, we refer to plot scores along the first PCA axis (soil PCA) as “soil gradient,” which was characterized by increased soil C, N, EC, pH, very fine sand, slit and clay, and soil water content following the sandy grassland restoration (Tables S1 and S2). Soil PCA was used to test how plant community‐average traits vary with soil gradient in the restoration of sandy grassland.

### 2.4 Plant functional traits

To measure the functional trait values of different plant communities along the entire gradient, we selected the 34 most abundant species (representing over 90% of plant biomass from 45 species), in which 6, 15, 22, and 20 occurred in mobile dune, semi‐fixed dune, fixed dune, and grassland, respectively. There were 4, 9, and 13 common species from the mobile dune to the semi‐fixed dune, from the semi‐fixed dune to the fixed dune, and from the fixed dune to the grassland, respectively. For species number, herbaceous plants dominated the four habitats of the sandy grassland (Table S3). Five to ten individuals of each species were collected in each quadrat to measure five plant traits: specific leaf area, (SLA), leaf dry matter content (LDMC), leaf carbon content (LCC), leaf nitrogen content (LNC), and plant height. These traits are related to resource use, leaf morphology, and plant size and are often used in studies of plant functional traits responses to environmental changes and their effects on ecosystem function (Jager, Richardson, Bellingham, Clearwater, & Laughlin, 2015; Lienin & Kleyer, 2012; Spasojevic, Grace, Harrison, & Damschen, 2014). Plant traits of all samples were determined using standard methodologies (Conti & Díaz, 2013; Cornelissen et al., 2003; Kichenin et al., 2013).

### 2.5 Data analysis

To characterize trait distributions in communities, we investigated both the abundance‐weighted and non‐weighted community‐average trait values (Kichenin et al., 2013) along the habitat or soil gradients of a sandy grassland restoration. Weighted community‐average trait values were calculated from the formula: WCA (trait_*X*_) = Σ*p*_*i*_ *x*_*i*_, where WCA (trait_*X*_) is the WCA for a *X* trait, *p*_*i*_ is the relative biomass of the *i*th species in the community, and *x*_*i*_ is the trait value of *i*th species. Non‐weighted community‐average trait values were calculated as the mean of all species in each plot (Mason et al., 2012). Linear regressions were performed between soil gradients and all weighted and non‐weighted community‐average traits.

Differences in the relative contributions of intra‐ and interspecific trait variation effects on the weighted and non‐weighted community‐averages along the restoration gradient of the sandy grassland were determined by the following methods (Kichenin et al., 2013; Lepš et al., 2011). The method is based on the decomposition of the total sum of squares (SS_specific)_ of the community‐level trait variance related to habitat changes into “interspecific” (SS_interspecific_), “intraspecific” (SS_intraspecific_), and “covariation” (SS_cov_) effects, such that SS_specific_ = SS_interspecific_ + SS_intraspecific_ + SS_cov_ (Kichenin et al., 2013; Lepš et al., 2011). First, we calculated the “specific” average and “interspecific” average traits in each plot. The “specific” plot‐average trait values were calculated using plant trait values as measured on that plot, which included both inter‐ and intraspecific effects. The “interspecific” plot average trait values were calculated using plant trait values averaged over all plots across the habitat changes, which was caused by differences of plant traits in species turnover. Then, we calculated the “intraspecific” plot average traits as the difference between specific and interspecific plot average trait values, by subtracting interspecific from specific. For each trait, we separately performed a one‐way ANOVA for specific, intraspecific, and intraspecific community‐averages with habitat as an explanatory variable. Finally, we calculated the SS_cov_ part, which represented the effect of covariation between inter‐ and intraspecific trait variation, by subtracting SS_interspecific_ and SS_intraspecific_ from SS_specific_. Consequently, the total variation in specific averages was regarded as 100% (e.g.). Based on the above, we could calculate the relative proportions of variability of interspecific, intraspecific, and covariation trait variation effects explained by habitat changes. We also determined the positive or negative responses of interspecific and intraspecific averages to habitat changes, depending on their contributions to total variation in specific averages. If both the effects of inter‐ and intraspecific trait variation positively contributed to the total variation in specific averages, then SS_specific_ would be higher than either of the two effects. However, if the two effects are opposite, then SS_specific_ would be lower than expected (Kichenin et al., 2013; Lepš et al., 2011).

The effect of habitat change on the interspecific, specific, and intraspecific plot averages for each trait was tested across the 24 plots by a one‐way ANOVA. Then, we extracted the sums of squares for each of the three plot‐average measures (SS_interspecific_, SS_specific_ and SS_intraspecific_) explained by habitat change. Finally, we calculated the variance partitioning of each trait across the plot level. All statistical analyses were carried out using SPSS (version 16.0). All functional diversity indices were calculated using the statistical package FDiversity v. 2011 (Casanoves et al., 2011).

## 3 Results

**Figure 1:**
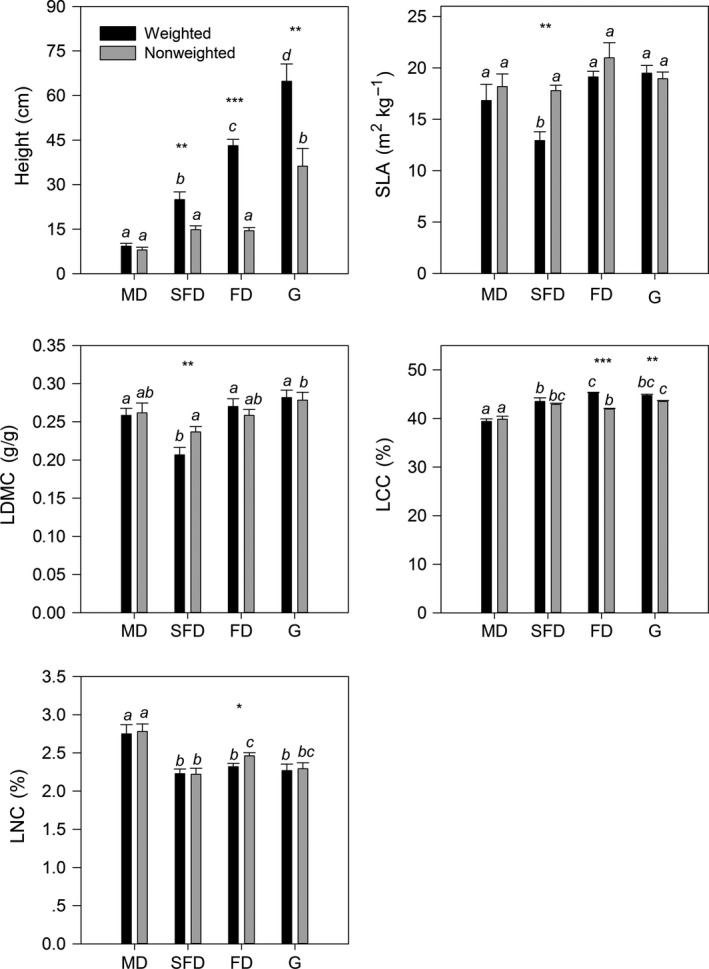
Comparisons of five weighted and non‐weighted community‐average trait values at four habitats of sandy grassland. Values represent means ± standard errors (*n* = 6). Different letters in from mean values indicate statistical difference among different habitats at, *p < .05, **p < .01,***p < .001. SLA, specific leaf area; LDMC, leaf dry matter content, LCC, leaf carbon content; LNC, leaf nitrogen content

Except for the non‐weighted community‐average of SLA, habitat changes had significant effects on five weighted and non‐weighted community‐average traits (Figure 1, *p* < .05). The weighted community‐averages of height and LCC increased following the restoration of sandy grassland. The weighted community‐averages of SLA and LDMC in the semi‐fixed dune were lower than in the mobile dune, the fixed dune, and the grassland. The weighted community‐averages of LNC in the mobile dune were higher than in the semi‐fixed dune, the fixed dune, and the grassland. The non‐weighted community‐average of height in the grassland was higher than in the mobile dune, the semi‐fixed dune, and the fixed dune. We also found some differences between weighted and non‐weighted community‐average traits in the same habitat, for example, the higher weighted community‐average of height in the semi‐fixed dune, the fixed dune, and the grassland, the higher weighted community‐average of LCC in the fixed dune and the grassland; the lower weighted community‐averages of SLA and LDMC in the semi‐fixed dune, and the lower weighted community‐average of LNC in the fixed dune (Figure 1). So, five weighted community‐average traits had the strongest responses to habitat changes in the restoration of the sandy grassland.

**Figure 2:**
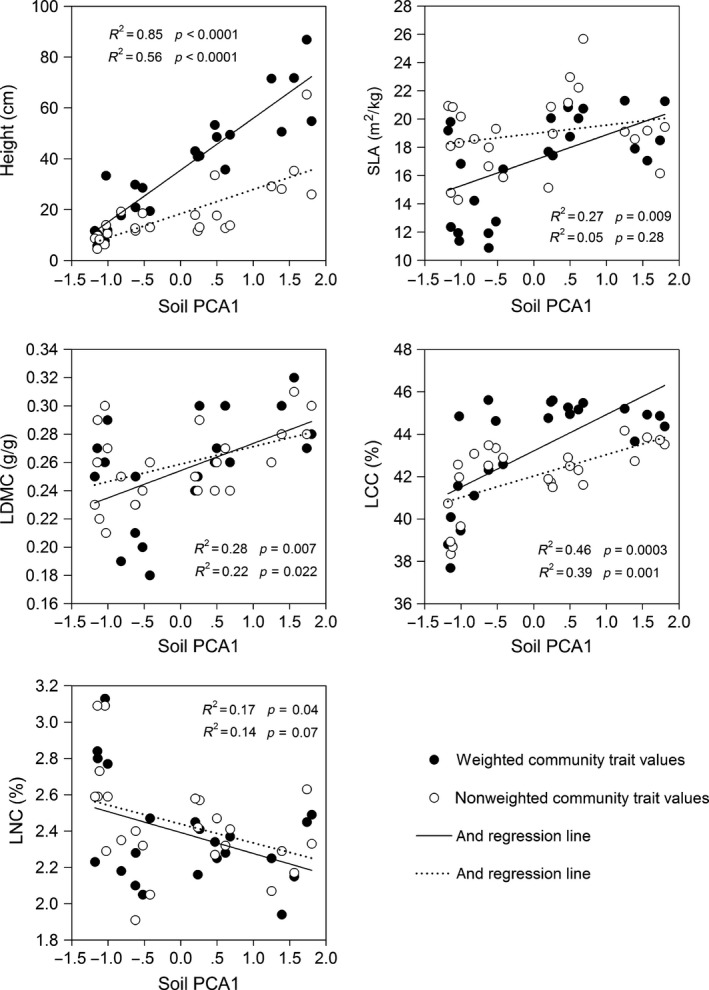
Responses of five weighted and non‐weighted community‐average trait values to a soil gradient. Regression coefficients (*R* ^2^) and *p* values are given for generalized linear model regressions of weighted and non‐weighted community‐level average values by soil gradient. SLA, specific leaf area; LDMC, leaf dry matter content; LCC; leaf carbon content; LNC, leaf nitrogen content

Soil gradient significantly explained most of the weighted and non‐weighted community‐average traits (Figure 2, *p* < .05). Weighted community‐averages of height, SLA, LDMC, and LCC significantly increased with soil gradient (*p *<* *.05). Similarly, non‐weighted community‐averages of height, LDMC, and LCC also significantly increased with soil gradient (*p *<* *.05). Weighted and non‐weighted community‐average of LNC significantly decreased with soil gradient (*p *<* *.05). Non‐weighted community‐average of SLA was independent of soil gradient (*p *>* *.05).

We also found that habitat changes strongly affected the specific averages of five weighted and non‐weighted community‐level traits (Table 1, *p* < .01). Regarding plant height, SLA, LCC, and LNC, habitat changes had significant effects on the weighted interspecific and intraspecific averages (*p *<* *.001). Similarly, habitat changes also significantly affected the non‐weighted interspecific and intraspecific averages of height, LCC, and LNC (*p *<* *.01). For LDMC, habitat changes also had significant effects on the interspecific averages at weighted and non‐weighted community level (*p *<* *.05).

**Table 1:** Results of one‐way ANOVAs from habitat change effects on five weighted and non‐weighted community‐average trait values. Interspecific, specific, and intraspecific variability effects were separately analyzed by one‐way ANOVA. Sum of squares (SS) correspond to the amount of variability

	Interspecific					Specific					Intraspecific				
	SS	*df*	MS	*F*	*p*	SS	*df*	MS	*F*	*p*	SS	*df*	MS	*F*	*p*
*Weighted community‐averages*															
Height	8125.17	3	2708.39	95.37	< .001	9770.58	3	3256.86	93.04	< .001	1180.80	3	393.60	84.58	< .001
SLA	197.10	3	65.70	21.89	< .001	195.64	3	65.21	33.56	< .001	19.05	3	6.35	16.11	< .001
LMDC	0.01	3	0	16.47	< .001	0.02	3	0.01	21.56	< .001	0	3	0.00	3.07	.051
LCC	69.51	3	23.17	23.81	< .001	125.20	3	41.73	40.63	< .001	11.10	3	3.70	16.03	< .001
LNC	0.79	3	0.26	11.16	< .001	1.41	3	0.47	24.15	< .001	0.15	3	0.05	23.95	< .001
*Non‐weighted community‐averages*															
Height	1184.15	3	394.72	13.23	< .001	2425.20	3	808.40	25.65	< .001	286.43	3	95.48	11.37	< .001
SLA	15.70	3	5.24	2.30	.108	37.02	3	12.34	8.74	< .01	44.23	3	14.74	7.58	< .01
LMDC	0.01	3	0	6.65	< .001	0.01	3	0	7.39	< .01	0	3	0.00	1.40	.272
LCC	11.48	3	3.83	11.63	< .001	46.64	3	15.55	29.46	< .001	14.60	3	4.87	94.97	< .001
LNC	0.48	3	0.16	12.04	< .001	1.11	3	0.37	29.69	< .001	0.22	3	0.07	43.05	< .001

SLA, specific leaf area; LDMC, leaf dry matter content; LCC, leaf carbon content; LNC, leaf nitrogen content.

**Figure 3:**
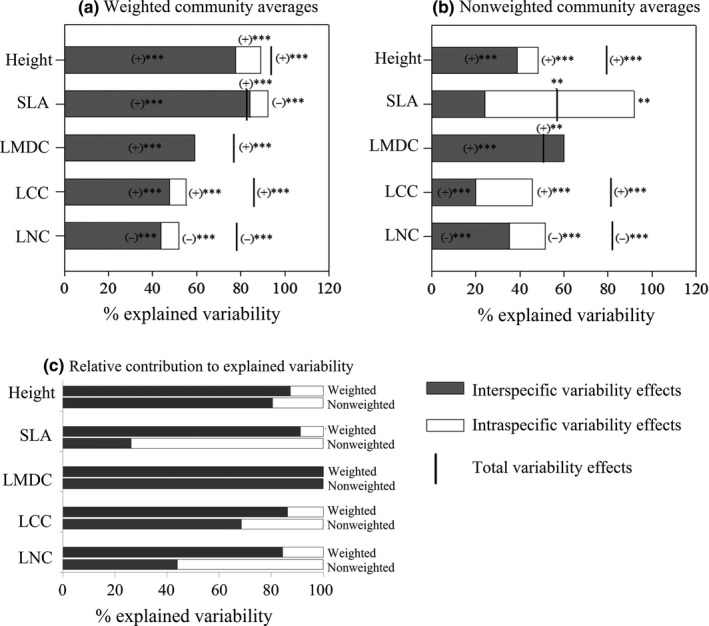
Decomposition of the total variability in community‐average trait values explained by habitat changes into interspecific, intraspecific, and covariation effects for (a) weighted and (b) non‐weighted community‐average trait values; and (c) overview of the relative contributions of inter‐ and intraspecific variability effects to the explained variability for five traits. SLA, specific leaf area; LDMC, leaf dry matter content, LCC, leaf carbon content; LNC, leaf nitrogen content. Covariation strength is represented by the interval between the “total variability” and the sum of inter‐ and intraspecific variability effects. The signs (±) and statistical significance (**p <* *.01,***p <* *.001) of interspecific, intraspecific, and total variability effects are presented on the graph when significant. Total variability value that is higher than the sum of inter‐ and intraspecific variability suggests positive covariation, and total variability value that is lower than the sum of inter‐ and intraspecific variability effects indicates negative covariation

The contribution of interspecific variation in explaining the response of weighted community‐average trait variation to habitat changes was significant for each of the five traits (Figure 3a). In contrast, the effect of intraspecific variation was significant for height, SLA, LCC, and LNC. There was a positive covariation between the effects of inter‐ and intraspecific variation on community‐average trait values for height, LDMC, LCC, and LNC, whereas a negative covariation occurred for SLA (Figure 3a). The negative covariation for weighted community‐averages of SLA indicated a decrease in intraspecific SLA of dominant species with habitat changes.

The contribution of inter‐ and intraspecific variation in explaining the response of non‐weighted community‐average trait variation to habitat changes was significant for height, LCC, and LNC (Figure 3b). Additionally, there was a strong positive covariation between the effects of inter‐ and intraspecific variation on non‐weighted community‐average trait values for height, LCC, and LNC.

More importantly, the relative contribution of inter‐ vs. intraspecific variation in explaining the community‐average trait variation with habitat changes was consistently higher for weighted than for non‐weighted averages for height, SLA, LCC, and LNC (Figure 3c). Thus, changes in the weighted community‐averages of these traits with habitat changes were primarily determined by interspecific variation (Figure 3c).

## 4 Discussion

This study is one of the first to identify the responses of abundance‐weighted and non‐weighted community‐average traits to habitat changes and the relative contribution of interspecific and intraspecific trait variation to the effects on community‐average trait values in a sandy grassland ecosystem. Overall, our findings agreed with the initial hypotheses. For all five functional traits, the effect of interspecific variation was much greater than that of intraspecific variation on the response of the community‐average traits to habitat changes in the restoration of sandy grassland. This is consistent with other studies where the response of a plant community to an environmental gradient is primarily driven by the interspecific trait variation (Albert et al., 2011; Kichenin et al., 2013; Vilà‐Cabrera et al., 2015). Comparative analyses between abundance‐weighted and non‐weighted average variations confirm the great contribution of dominant species in intraspecific variation to community‐level averages across habitat changes in the restoration of a sandy grassland.

We found the contrasting patterns of five weighted community‐average traits in response to habitat changes in the restoration of a sandy grassland. The increase in weighted community‐average traits such as height and LCC with habitat restoration suggests that the high‐standing plants with high leaf C content dominate the habitat following the restoration of a sandy grassland. This can be explained by the increasing supply of soil resources following habitat restoration, which facilitates herbaceous plants with a fast‐growth strategy to become dominant species in the restoration of a sandy grassland. This also supports the finding that the local dominance of relatively taller plants can lead to higher plant biomass, thus promoting greater C storage in plants (Conti & Díaz, 2013). However, there was a greater weighted community‐averaged of leaf N content in mobile dunes in comparison to other habitats, suggesting that the sand pioneer species, *A. squarrosum* could adapt to the extreme environment with barren soil conditions by means of higher leaf N content. Species growing in nutrient‐poor environments tend to maintain a high plant N concentration in order to complete their life history in time (Chapin, 1980). Semi‐fixed dunes had lower weighted community‐average values of SLA and LDMC compared to other habitats, suggesting that the asexual reproduction shrub, *A. halodendron* could adapt to semi‐fixed dunes (the relative barren and dry soils) through relatively lower SLA and LDMC values. This may be reflected in the shrub *A. halodendron,* which tends to be productive and has a relatively high investment to leaf structure “defenses” in living conditions of wind erosion and sand bury (Cornelissen et al., 2003).

We also observed the contrasting responses of five weighted community‐average traits to soil gradient in the restoration of a sandy grassland. Weighted community‐averages of height, SLA, LDMC, and LCC increased with the soil gradient, while LNC decreased. This is in agreement with other studies showing that a soil gradient may induce the coordinated responses of multiple functional traits (Bernard‐Verdier et al., 2012; Jager et al., 2015; Moraes et al., 2016). These results suggest that species having fast‐growing strategies (i.e., high SLA, plant height, and LCC) are firmly associated with increased soil resource availability following the restoration of a sandy grassland. However, species growing in extremely barren soils are significantly tended to maintain the higher leaf N content. These results provide evidence that a soil gradient controlled by environmental stress was restricted to species selection and survival in sandy grassland ecosystems.

In our study, weighted interspecific variance of five functional traits was between 44% and 84% of the total variance of weighted community‐averages and was more than 56% for height, SLA, and LDMC. The dominance of interspecific trait variation for all five traits suggests that when a trait variation comes primarily from interspecific differences, then the variation in community‐level traits across habitat changes is generated primarily through changes in the relative abundances of species over habitat changes following the restoration of a sandy grassland (Kichenin et al., 2013; Lepš et al., 2011). However, the weighted intraspecific variance of height, SLA, LCC, and LNC also contributed to 8%–11% of the total variance of weighted community‐averages. Non‐weighted intraspecific variance of SLA and LCC was higher than that of non‐weighted interspecific variance and contributed to 68% and 25% of total variance of non‐weighted community‐averages. Therefore, the effect of intraspecific trait variation on community‐level traits should be considered, which is in agreement with other findings that the intraspecific trait variation plays a role in the process of plant community assembly (Carlucci et al., 2015; Kichenin et al., 2013; Laughlin et al., 2012; Siefert, 2012; Vilà‐Cabrera et al., 2015). This suggests that neglecting intraspecific trait variance will lead to the underestimation of community‐level traits responses to environmental changes (Auger & Shipley, 2013; Lepš et al., 2011).

The positive relationships with soil gradient observed for the weighted and non‐weighted community‐averages of height and LCC were mainly driven by a substantial positive covariation. The negative relationship with soil gradient observed for the weighted community‐average of LNC was also driven by a substantial positive covariation. Further, the negative covariation between inter‐ and intraspecific variation of weighted community‐average responses to habitat changes contributed to the total variation for SLA. This similar pattern is reported by other studies (Kichenin et al., 2013; Poorter, Niinemets, Poorter, Wright, & Villar, 2009). The interspecific increase and intraspecific decrease in SLA with habitat changes may be explained by the fact that increasing soil resource availability induces an increase in relative abundance of dominant species with higher SLA and a decrease in intraspecific SLA of these species following vegetation succession in the restoration of a sandy grassland. These results agree with the findings that although the direct effect of intraspecific trait variation on the response of a community‐level trait to habitat changes is relatively low, its indirect effect through positive or negative covariation with interspecific trait variation is substantial (Kichenin et al., 2013).

## 5 Conclusion

Our results have clearly illustrated the relative contribution of the effects of interspecific, intraspecific trait, and their covariations on the response of community‐average trait values to restoration of a sandy grassland ecosystem. Community‐level trait values with habitat changes in the sandy grassland restoration are dominated by the interspecific trait variation. The intraspecific trait variation and their covariations with interspecific trait variation contribute to community‐level trait responses to habitat changes. It was suggested that changes in vegetation dynamics and ecosystem function following the sandy grassland restoration will depend on the interspecific, intraspecific variation, and their roles in shaping plant adaptation and community responses to habitat changes. It is therefore an essential step in examining the role of these two components of community structure in the response of a plant community to environmental changes toward linking environmental changes to changes in species composition and ecosystem function. Furthermore, the intraspecific trait variation information should not be neglected in small or short‐term environmental changes. The relative contribution of intraspecific trait variation and its covariation with interspecific variation to changes of community‐level traits can improve our mechanistic understanding of community assembly in environmental changes.

## Conflict of Interest

None declared.
