# Supplementary material for: Contrasting effects of plant inter‐ and intraspecific variation on community trait responses to restoration of a sandy grassland ecosystem
Source: Ecol Evol. 2017 Jan 24;7(4):1125–34. doi: 10.1002/ece3.2711 (PMC5306005; doi:10.1002/ece3.2711)
Supplement: Supplementary file 1 [file ECE3-7-1125-s001.docx]

Table S1. Vegetation characteristics and soil properties at four habitats of sandy grassland (Mean ± SE, n=6).

|  | MD | SFD | FD | G | F | *P* |
| --- | --- | --- | --- | --- | --- | --- |
| ***Vegetation characteristics*** | | | | | | |
| Cover (%) | 3.78±0.39 ^a^ | 44.37±1.50^b^ | 65.53±2.96 ^c^ | 72.00±2.15^d^ | 240.45 | < 0.001 |
| Species richness | 1.83±0.17^a^ | 6.53±0.59^b^ | 10.23±0.44^c^ | 7.47±0.60^b^ | 52.33 | < 0.001 |
| Biomass( g m^-2^) | 2.20±0.39^a^ | 98.10±7.27^b^ | 131.41±8.20^c^ | 190.38±19.15^d^ | 50.93 | < 0.001 |
| ***Soil properties*** | | | | | | |
| C (g kg^-1^) | 0.43±0.01^a^ | 1.10±0.08^b^ | 3.94±0.19^c^ | 5.17±0.29^d^ | 160.50 | < 0.001 |
| N (g kg^-1^) | 0.11±0.01^a^ | 0.15±0.01^a^ | 0.47±0.02^b^ | 0.61±0.03^c^ | 122.00 | < 0.001 |
| C/N | 4.23±0.43^a^ | 7.56±0.30^b^ | 8.48±0.21^c^ | 8.48±0.12^d^ | 49.16 | < 0.001 |
| pH | 7.39±0.02^a^ | 7.50±0.04^a^ | 8.01±0.10^b^ | 8.47±0.06^c^ | 58.02 | < 0.001 |
| Bulk density (g cm^-3^) | 1.62±0.02^a^ | 1.57±0.01^a^ | 1.50±0.02^b^ | 1.33±0.04^c^ | 26.38 | < 0.001 |
| Electricity conductivity (μs cm^-1^) | 7.83±0.49^a^ | 11.23±0.98^a^ | 20.93±1.71^b^ | 30.00±3.41^c^ | 25.47 | < 0.001 |
| Coarse sand (2-0.25 mm, %) | 60.68±2.03^a^ | 47.01±1.55^b^ | 43.65±1.65^b^ | 21.33±4.72^c^ | 33.82 | < 0.001 |
| Fine sand (0.25-0.1 mm, %) | 37.49±2.12^ac^ | 49.73±1.31^b^ | 42.22±3.16^a^ | 35.22±2.35^c^ | 7.57 | < 0.01 |
| Very fine sand (0.1-0.05 mm, %) | 1.48±0.09^a^ | 1.85±0.27^a^ | 5.62±1.32^a^ | 31.36±5.43^b^ | 26.15 | < 0.001 |
| Silt + Clay (<0.05 mm, %) | 0.35±0.03^a^ | 1.41±0.23^a^ | 8.51±0.61^b^ | 12.09±1.77^c^ | 35.90 | < 0.001 |
| Soil water content (%) | 2.86±0.17^a^ | 2.60±0.28^a^ | 3.36±0.51^a^ | 5.01±0.53^b^ | 7.23 | < 0.01 |

The results are based on One-way ANOVAs from habitat changes effects.

Table S2. Intra-set correlations of the soil properties, eigenvalue and cumulative percentage variance for the first two axes of principal component analysis (PCA).

|  | PCA1 | PCA2 |
| --- | --- | --- |
| C | 0.98^***^ | 0.16 |
| N | 0.97^***^ | 0.12 |
| C/N | 0.74^***^ | 0.46^*^ |
| pH | 0.94^***^ | 0.08 |
| Bulk density | -.90^***^ | 0.11 |
| Electricity conductivity | 0.93^**^ | 0.04 |
| Coarse sand (2-0.25 mm) | -0.90^**^ | 0.10 |
| Fine sand (0.25-0.1 mm) | -0.43^*^ | 0.56^**^ |
| Very fine sand (0.1-0.05 mm) | 0.87*** | -0.44* |
| Silt + Clay (<0.05 mm) | 0.96*** | 0.08 |
| Soil water content | 0.75*** | -0.27 |
| Eigenvalues | 0.87 | 0.08 |
| Cumulative percentage variance (%) | 86.80 | 95.00 |

Table S3. Vegetation species composition, life-group type and important values (%) among different sandy habitats

| Species name | Life-group | Important value （%） | | | |
| --- | --- | --- | --- | --- | --- |
|  |  | MD | SFD | FD | SG |
| *Artemisia frigida* | PF |  |  |  | 0.79 |
| *Pennisetum centrasiaticum* | PG |  |  | 0.21 | 7.54 |
| *Melissitus ruthenicus* | PL |  |  | 0.87 |  |
| *Cleistogenes squarrosa* | PG |  |  | 6.08 | 4.05 |
| *Artemisia halodendrom* | S |  | 46.83 |  |  |
| *Lespedeza davurica* | S |  | 0.17 | 1.67 | 4.26 |
| *Corispermum macrocarpum* | AF |  | 28.45 | 0.24 |  |
| *Delphinium grandiflorum* | PF |  |  |  | 0.31 |
| *Artemisia sieversiana* | AF |  |  |  | 6.21 |
| *Euphorbia humifusa* | AF |  | 1 | 0.18 | 0.13 |
| *Cynanchum theisiodes* | PF |  | 3.42 |  | 0.21 |
| *Potentilla bifurca* | AF |  |  |  | 0.72 |
| *Setaria viridis* | AG | 35.25 | 8.54 | 2.15 | 1.21 |
| *Eragrostis pilosa* | AG |  |  | 0.36 |  |
| *Artemisia scoparia* | AF |  |  | 65.28 | 53.64 |
| *Salix gordejevii* | S | 1.78 |  |  |  |
| *Tribulus terretris* | AF | 1.14 |  | 0.79 | 0.09 |
| *Chenopodium acuminatum* | AF |  | 0.47 | 1.98 | 3.25 |
| *Sonchus oleraceus* | AF |  | 0.51 |  |  |
| *Ixeris denticulata* | AF |  | 2.63 |  |  |
| *Leymus secalinus* | PG |  |  |  | 4.75 |
| *Artemisia frigida* | S |  |  | 1.99 |  |
| *Phragmites communis* | PG | 0.9 | 0.17 | 0.42 | 9.46 |
| *Erodium stephanianum* | AF |  |  | 1.5 | 0.6 |
| *Digitaria cilliaris* | AG | 0.84 | 1.14 |  |  |
| *Atraphaxis manshurica* | S |  |  | 2.85 |  |
| *Allium mongolicum* | PF |  |  | 4.03 | 0.62 |
| *Agriophyllum squarrosum* | AF | 55.27 |  |  |  |
| *Echinops gmelini* | AF |  | 1.71 | 4.93 | 0.92 |
| *Tragus berteronianus* | AF |  |  | 0.42 |  |
| *Bassia dasyphylla* | AF |  | 1.28 | 0.71 |  |
| *Gueldenstaedtia stenophylla* | PF |  |  | 0.19 |  |
| *Caragana microphylla* | S | 4.82 | 2.61 | 2.06 |  |
| *Salsola collina* | AF |  | 1.07 | 1.09 | 1.24 |
| Species number |  | 7 | 15 | 22 | 20 |

MD, Mobile dune; SFD, Semi-fixed dune; FD, Fixed dune; G, Grassland; The specie dominance (DV) in each habitat was calculated using the ordinary formula DV = (RW + RH + RC)/3, where RA is the relative biomass, RH is the relative height, and RC the relative cover of the species. RW, RH, and RC were all represented as percent values. AF Annual forbs, AG annual grass, PF, perennial forbs, PG，perennial grass, S shrub.
